# Stripe pattern differences can be used to distinguish individual adult zebrafish

**DOI:** 10.1371/journal.pone.0311372

**Published:** 2024-10-01

**Authors:** Shinichi Meguro, Takahiro Hasumura

**Affiliations:** 1 Biological Science Research, Kao Corporation, Tochigi, Japan; 2 Human Health Care Product Research, Kao Corporation, Tokyo, Japan; University of Naples Federico II: Universita degli Studi di Napoli Federico II, ITALY

## Abstract

Adult zebrafish are commonly used as disease models in biomedical research, but unlike in other model organisms such as rodents, there is no simple method for distinguishing individuals, even though the importance of individual differences is recognized in such research. We developed a side viewing device that can be used to capture images of stripe patterns and identified eight distinct components of stripe patterns on the caudal and anal fins that allowed us to distinguish individual fish. We found that the stripe patterns were consistent for at least 8 weeks in males and females of two lines of wild-type zebrafish. These results suggest that individual adult zebrafish can be distinguished in an easy and non-invasive manner, allowing researchers to incorporate individual differences in biomedical research as in rodent models.

## Introduction

Zebrafish (*Danio rerio*) have become increasingly popular as experimental animals because their organs and tissues are similar to those of humans in both structure and function. In addition, zebrafish have many advantages as model organisms for investigating pathological conditions in humans; for example, they are easy to breed and manipulate genetically and are inexpensive to maintain, unlike mammals [1]. Zebrafish have been used in biomedical research to investigate diabetes, cancer, and neurodegenerative and cardiovascular diseases, among others [2–7]. It has also been shown that high-fat diets can induce body fat accumulation, hypercholesterolemia, and impaired cognitive function in zebrafish, and consecutive exercise can induce muscle hypertrophy [8–13]. Such studies often don’t consider the physiological effects on individual fish. If the effects could be followed in individual fish, however, our understanding of these physiological change issues could be advanced. It might also be possible to reduce the number of fish used in each study.

Zebrafish live in groups in the wild; in experimental situations, multiple zebrafish are placed in a single aquarium. Techniques for identifying and marking individual zebrafish have been used [14–17], but unlike the case of rodent models, in which individuals can be identified and tracked by using a variety of methods, identification of individual zebrafish is not well established in biomedical research [18]. A simple method for identification of individuals would greatly expand the utility of zebrafish as a model organism and improve its reliability in studying disease.

We hypothesized that stripe pattern differences could be used as a simple method to distinguish individual adult zebrafish. Although many reports detail the mechanism of stripe pattern formation, to our knowledge, no studies have investigated individual stripe pattern differences in detail as a method of individual identification [15, 19–21]. We designed a side viewing device that can be used to observe and document body and fin stripe patterns precisely. We used the device to examine the stripe pattern characteristics of caudal and anal fins of male and female zebrafish of two lines and determined that these patterns persist over at least 8 weeks and can be used for individual identification. Our findings offer a new method to distinguish individual adult zebrafish in a simple, reliable, and non-invasive manner.

## Materials and methods

### Ethics statement

All animal experiments were conducted in strict accordance with the regulations approved by the Animal Care and Experimentation Facility Committee of Kao Corporation (Tokyo, Japan) and with those outlined in *The Zebrafish Book* [22] and *Guide for the Care and Use of Laboratory Animals*, 8th edition [23]. This study was conducted as recommended by the ARRIVE guidelines.

### Animals

Two lines of wild-type adult zebrafish, RW (RIKEN, Wako, Saitama, Japan) and MS (Meito Suien Co., Ltd., Remix, Nagoya, Japan), were used. All fish were raised and maintained under a 14:10-h light: dark cycle at 28°C in recirculating aquaculture systems for zebrafish (Meito Suien Co., Ltd.). Water quality was ensured by monitoring and maintaining parameters in accordance with *The Zebrafish Book* [22]. The animals used in the experiments were all offspring of a single spawning event of each line. The fish were not bred during the study period. They were fed Otohime B2 (Marubeni Nisshin Feed Co. Ltd., Tokyo, Japan) as a standard fish diet, during the experimental period.

### Device for side viewing of adult zebrafish

**Fig 1:**
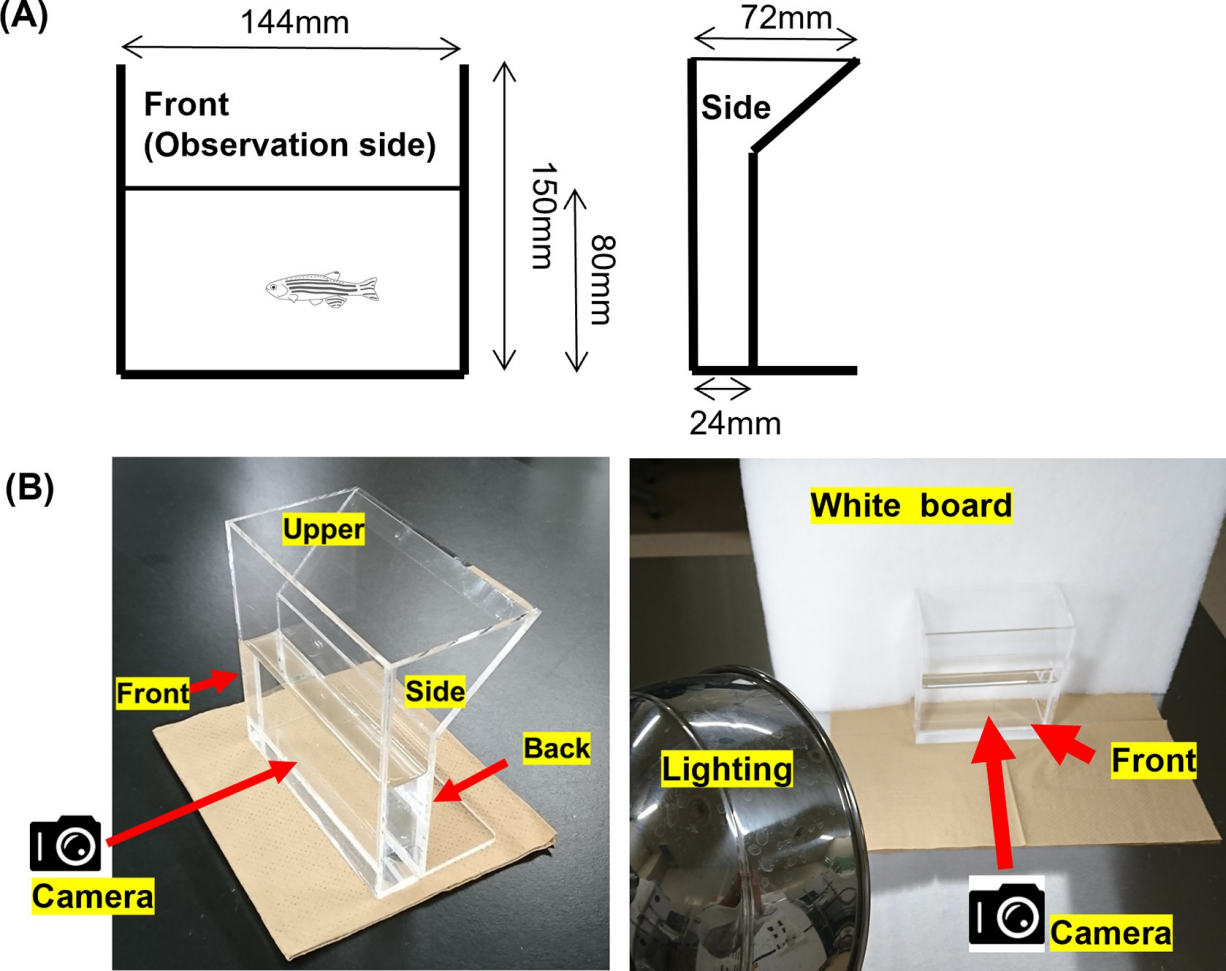
Transparent acrylic side viewing device. (A) The width of the side (24 mm) is no greater than the length of an adult zebrafish. Fish can swim freely back and forth, presenting a side view to the camera. (B) Photographs were taken from the front at 1/400 s. Oblique lighting was supplied from behind the camera.

The stripe patterns were observed and recorded by using the transparent side viewing device shown in Fig 1. The device is an acrylic aquarium (inside: 144 mm wide, 150 mm tall, and 24 mm thick, filled with water to 80 mm depth). The narrow thickness restricts swimming to a single plane, back and forth, leaving the fish always visible from the side. The water temperature is not controlled, but the water in it was taken from tanks held at 28°C, and the temperature of the room where it was used was 26°C, so the temperature in the device was 26–28°C.

### Photography of fin shape and stripe patterns

Individual zebrafish were moved gently from their storage tank to the side viewing device using polyester net (Slim-net Shinari-S; Sudo & Company Inc., Aichi, Japan) with a smooth surface and fine mesh to prevent damage to the fins. The fish were allowed to swim freely in the device. Images were captured when the fish was heading to the left and the fins were open. Images were captured with a high-sensitivity digital camera (EX-ZR1000; Cacio, Tokyo, Japan) at a shutter speed of 1/400 s. In addition to the room’s overhead lights, a direct light (LDA11N-G, 1520 lm; Toshiba, Tokyo, Japan) was placed obliquely about 1 m behind the camera to provide sufficient illumination (Fig 1B). Each image was immediately examined, and if fin undulations were evident, another was taken; as a result, 2–10 images of each zebrafish were taken. Images were deemed to be suitable when the photo showed a fin with a clear shape and stripe patterns. The fish were then returned to their tank using the same net.

### Experimental protocol

#### Experiment 1: Identification of characteristic stripe patterns

Female and male adult zebrafish (MS type, 5–8 months old) were used in Experiment 1 (*n* = 48); they were housed eight to a tank at 28°C. Each was placed into the side viewing device as described above, photographed, and returned to the tank. Images of the breast, ventral, dorsal, caudal, and anal fins were examined in detail by eye; images were chosen for analysis if there was a clear image of fins and any differences in stripe number and patterns. After clear photographic images were selected, they were analyzed to evaluate the differences in fins and stripe patterns to identify individual fish.

#### Experiment 2: Monitoring of caudal and anal fin stripe patterns over 8 weeks

Five adult zebrafish in each of four groups were placed in 1.7-L tanks (1 fish per tank) for 8 weeks at 28°C: female and male RW-type zebrafish (7 months old) and MS-type zebrafish (6 months old). Each fish was housed individually to identify any changes in its stripe pattern from week 0 to week 8. Each fish was photographed in side view at 0, 4, and 8 weeks. The obtained photographs were digitally processed to remove the background and increase the contrast, making it easier to analyze the stripe pattern of each individual. The caudal and anal fin stripe patterns were assessed by eye by two observers. At 8 weeks, both observers independently investigated any changes in fin stripe patterns over time. Observer 1 was in charge of the experiment. Observer 2 was chosen by Observer 1 as a reliable person who did not know the details of the experiment. Therefore, Observer 1 was not blinded, but Observer 2 was blinded to the experimental conditions.

## Results

### Experiment 1

**Fig 2:**
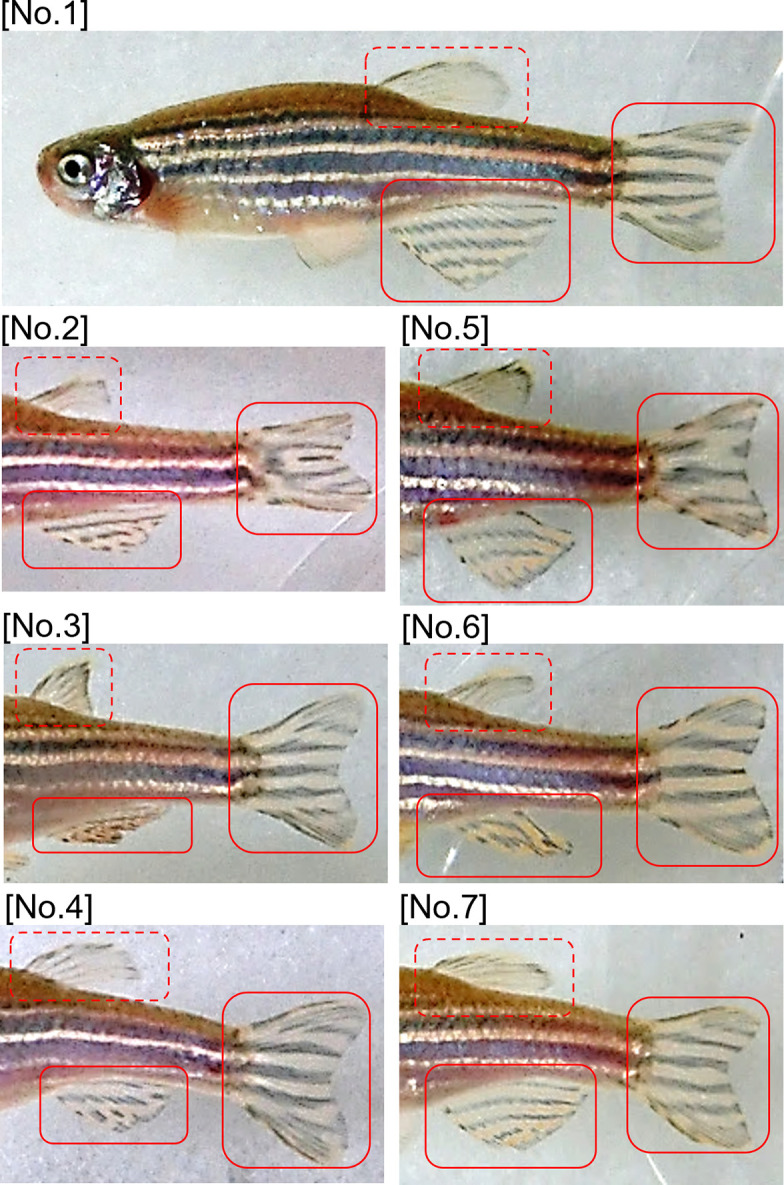
Side view images of seven adult zebrafish captured in the side viewing device. Solid red boxes delineate caudal and anal fins. Dashed red boxes delineate dorsal fins.

Side view images of adult zebrafish were easily and clearly captured in the side viewing device (Fig 1). Although we obtained clear images of the dorsal, caudal, and anal fins, we could not obtain clear images of the breast and ventral fins (Fig 2). We also examined the stripe patterns of the zebrafish trunk, which has five stripes, but found no distinctive differences among individuals. We found no individual differences in fin shape, and dorsal fin patterns did not show clear differences between individuals.

**Fig 3:**
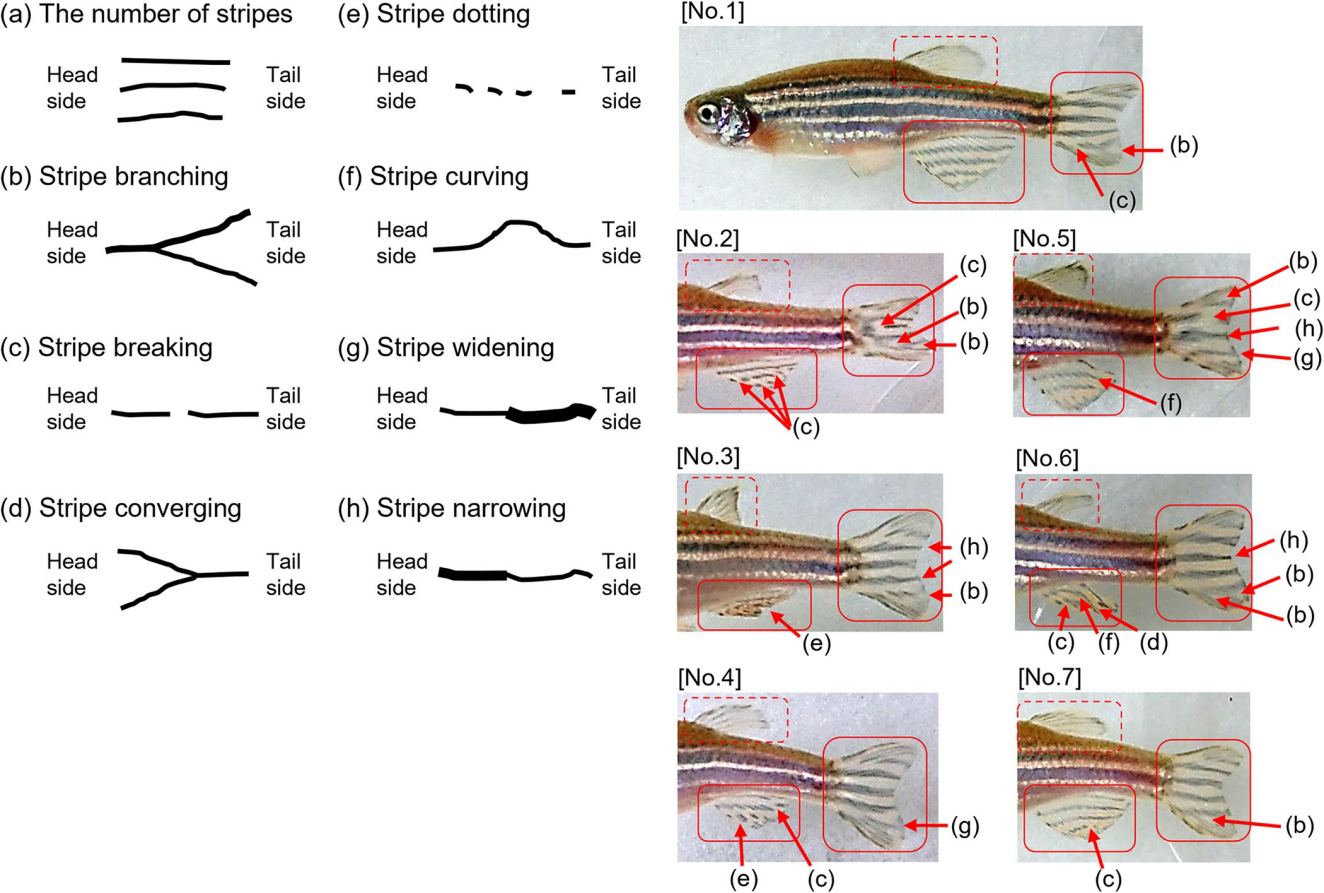
The eight components of stripe patterns observed on the caudal and anal fins of seven adult zebrafish. (a) Number of stripes, (b) branching (from 1 to >1 stripe), (c) breaking (≥1 gaps appear irregularly in a stripe), (d) converging (from >1 to 1 stripe), (e) dotting (where multiple dots appear on a stripe), (f) curving (a stripe curves to either side of the stripe), (g) widening (to at least double the original width), and (h) narrowing (to less than half of the original width).

The stripe patterns of the caudal and anal fins of each zebrafish were distinct. We defined eight components of stripe patterns, namely stripe number and seven differences in stripe shape (defined from head to tail): branching, breaking, converging, dotting, curving, narrowing, and widening (Fig 3). We observed no fish with the same stripe patterns. These results suggest that examining these eight components of stripe patterns in the caudal and anal fins would allow us to easily distinguish individual adult zebrafish.

### Experiment 2

**Fig 4:**
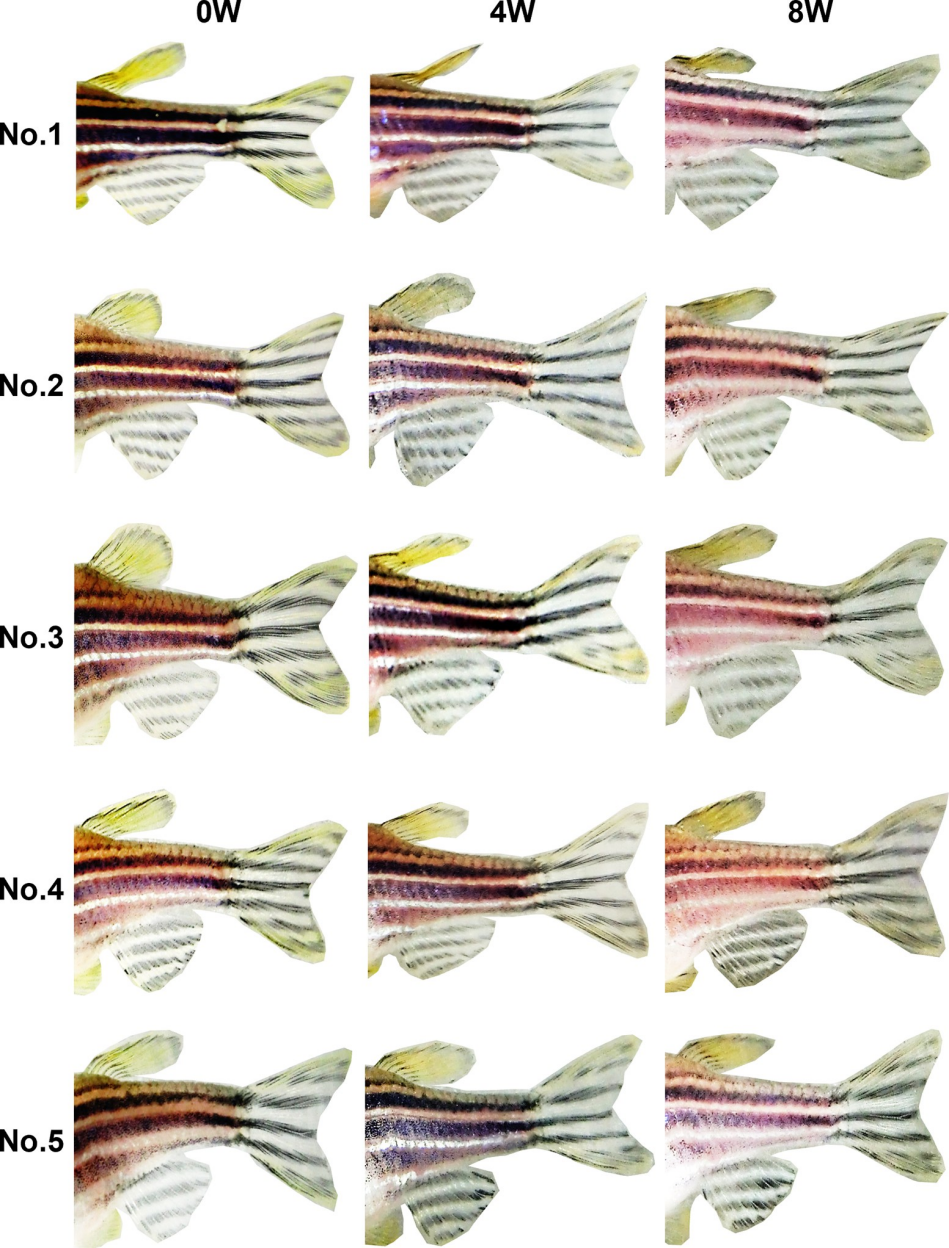
Stripe patterns of female RW zebrafish (*n* = 5) at 0, 4, and 8 weeks.

**Fig 5:**
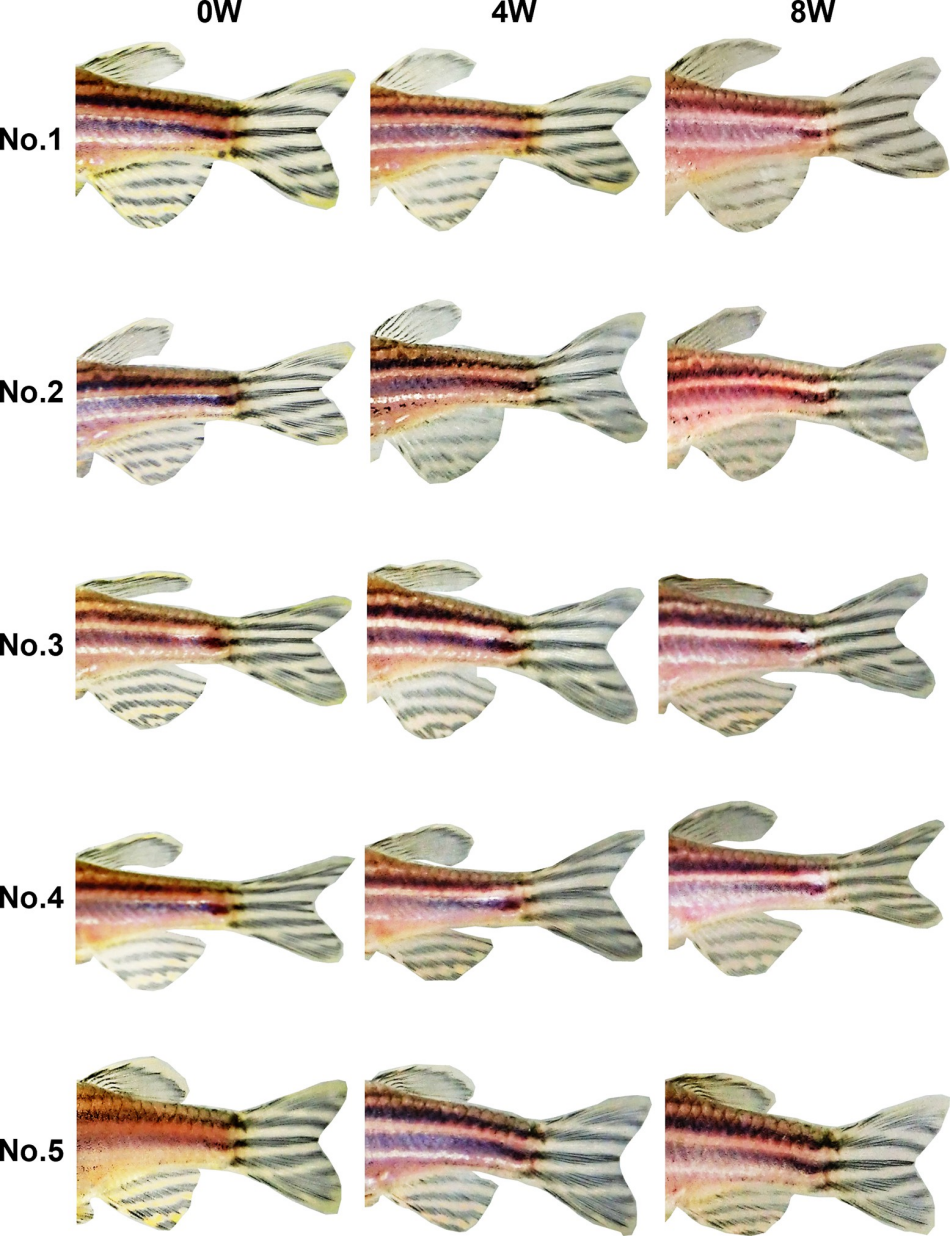
Stripe patterns of male RW zebrafish (*n* = 5) at 0, 4, and 8 weeks.

**Fig 6:**
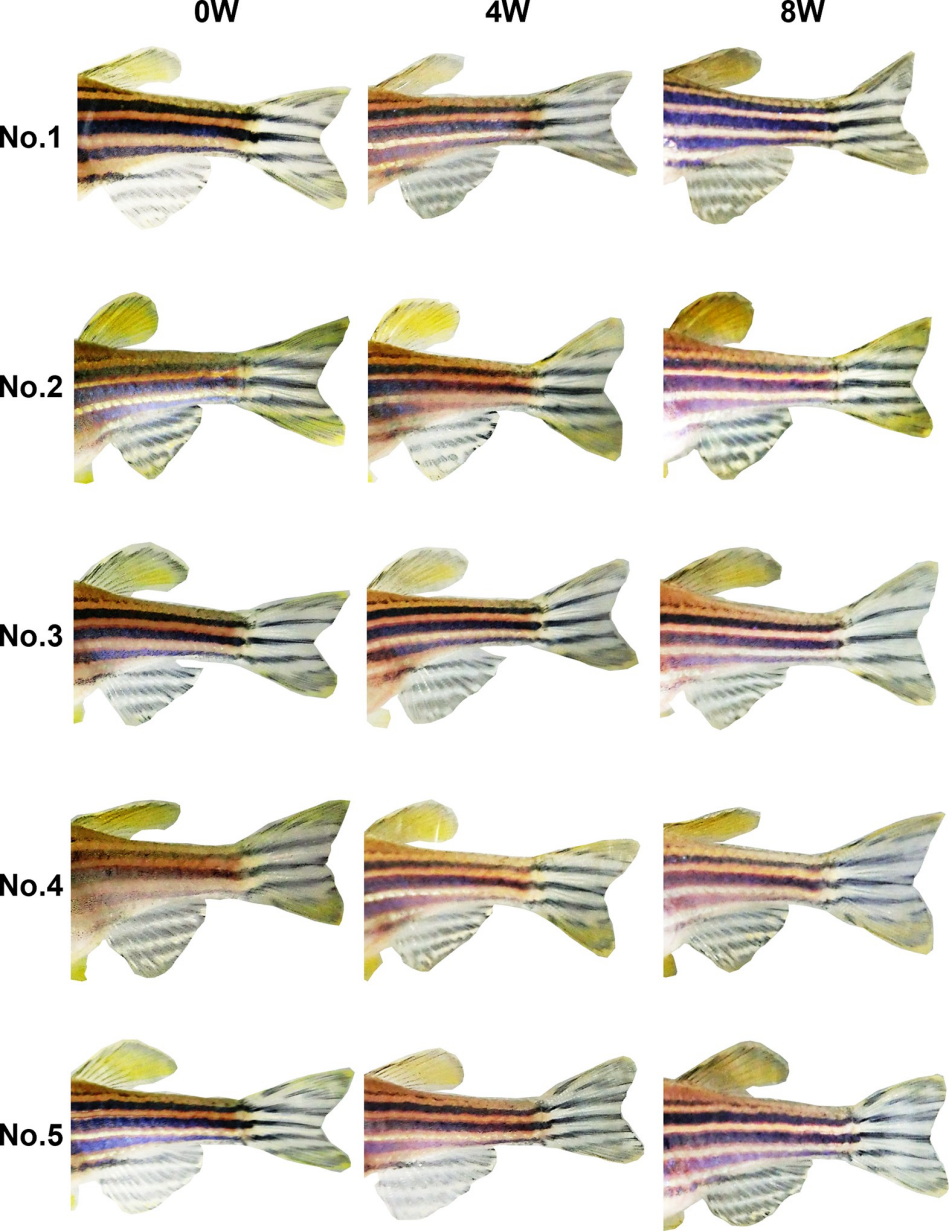
Stripe patterns of female MS zebrafish (*n* = 5) at 0, 4, and 8 weeks.

**Fig 7:**
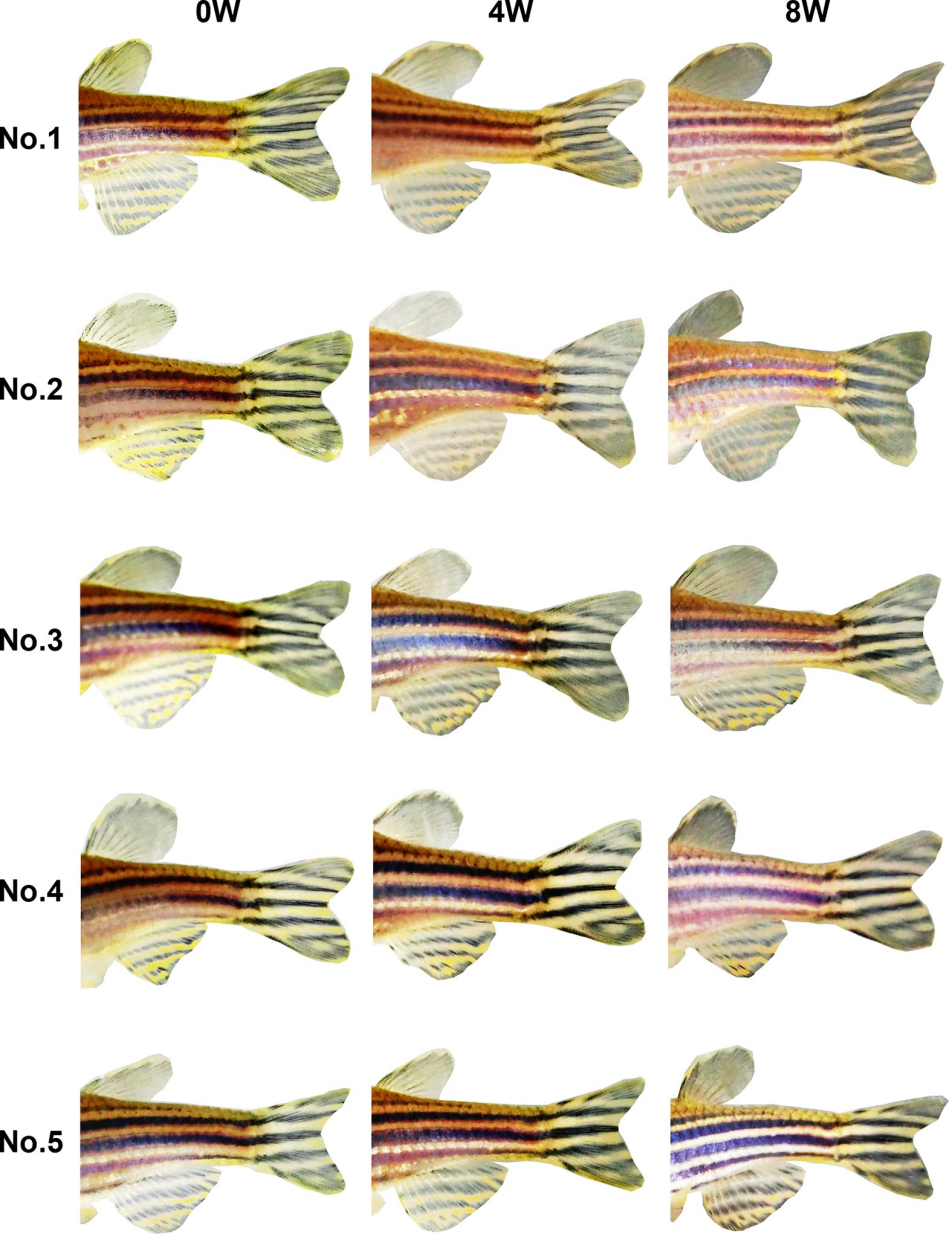
Stripe patterns of male MS zebrafish (*n* = 5) at 0, 4, and 8 weeks.

In week 0 photos, both observers were able to easily identify individual fish by using a combination of the eight stripe pattern components defined in Experiment 1 (Fig 3). At 4 and 8 weeks, no changes were evident in the stripe patterns of either the RW (Figs 4 and 5) or MS (Figs 6 and 7) fish. The results indicate that stripe patterns can be used to identify individual zebrafish in an experimental setting over a period of at least 8 weeks.

## Discussion

We demonstrated that (1) the stripe patterns of caudal and anal fins differ among individual adult zebrafish, (2) these differences can be confirmed non-invasively, and (3) they are maintained for at least 8 weeks. Biomedical research using adult zebrafish is often conducted over several weeks [2–13]. Therefore, it is important to be able to individuals when using zebrafish over several weeks. Studies that tracked individual identification over several weeks all used invasive methods: [15–17]: marking the caudal fin with a pencil-type soldering iron [16]; and subcutaneous injection of dye without significant alteration of behaviors [17]. As far as we know, however, our method is the only one that makes it possible to easily and non-invasively identify individual zebrafish for at least 8 weeks.

We found that individuals can be identified by differences in the stripe patterns of the caudal and anal fins. Stripes are larger and clearer on the trunk than on the fins, so if a stripe differs from individual to individual, the trunk stripe(s) might be useful to identify individuals. In fact, there may be individual differences in the stripe pattern of the trunk of AB wild-type zebrafish, but those on the left and right sides of each individual differ [15]. Therefore, this method requires extra care. But the caudal and anal fin are transparent, so unlike the trunk, there is no difference between the sides. Therefore, it might be more advantageous to use the stripe pattern in the fins to distinguish individual fish.

Eight components of the caudal and anal fin stripe patterns were obtained by analyzing stripe patterns of MS-type zebrafish for individual identification (Fig 3). These components could also be used to identify 10 RW and 10 MS individuals over a period of at least 8 weeks. It is unclear whether the eight components can be used to identify individuals in longer-term studies or in studies using more individuals. Furthermore, it is unclear whether other wild types (e.g., AB and TU) can be used in the same way. This is an important issue for future consideration.

It is important to understand the treatment effects in each fish during a study in biomedical research using zebrafish. However, as zebrafish usually lives in groups, they may influence each other during experiments. On the other hand, a group-living situation is ethically important when using adult zebrafish in experiments [22, 23]. Therefore, if it is possible to identify individual zebrafish, treatment effects on each individual within a test group can be understood rather than on the test group as a whole, which might advance biomedical research using zebrafish.

Analysis by repeated-measures analysis of variance (ANOVA) can be conducted only from data of individual fish during a study [24]. In addition, delta values in each treatment group during a study can be obtained from the change values of individual fish, as well as the absolute value in each treatment group. Accordingly, with the use of individual identification methods, statistical analyses can be performed with the same accuracy as they are in rodent studies, for which they have been established [16]. Because zebrafish do not have well-established genetically uniform inbred strains, unlike rodents, individual differences may be greater in zebrafish than in rodents. Therefore, it is even more important to be able to compare delta values in each fish for a detailed understanding of the treatment effect during a study. If the treatment effect can be examined as described above, our understanding of the differences in treatment effect between test groups will advance, leading to a reduction in the number of zebrafish required in test groups. Overall, the adoption of our new identification method may not only contribute to improved accuracy in biomedical research using zebrafish, but also improve the ethical treatment of the animals.

There are some limitations in using our method. The method cannot be used with zebrafish that do not have a stripe pattern on their caudal and anal fins (e.g., the transparent type or the leopard type) [20, 25, 26]. In addition, if the caudal and anal fins are injured or damaged, it may become difficult to distinguish differences. However, caudal (but not anal) fins have regenerated with the same stripe pattern after amputation [27]. Therefore, even if both the caudal and anal fins are lost during a study period, individual identification may be still possible by the stripe pattern of the regenerated caudal fin, although the accuracy of identification will be lower. Furthermore, zebrafish are frequently used for regeneration experiments using the caudal fin [28]. In that case, since part of the caudal fin is removed, the evaluation would use the stripe pattern of the caudal fin and anal fin up to the part of the caudal fin that regenerated after the start of the study, so the accuracy of individual identification also will be slightly lower. Finally, although we confirmed no changes in the stripe patterns of the caudal and anal fins in male and female individuals of two lines of wild-type zebrafish for up to 8 weeks, additional research is needed on other types of zebrafish and for longer periods to improve the usability of the method.

The process of removing a single fish from the holding tank, placing it in the side viewing device, photographing it until a picture suitable for image analysis is obtained, and then returning the fish to the tank took 2–3 min per fish, but it is as yet unclear whether or how this method improves work efficiency, workload, and individual identification accuracy over previous methods [14–17]. In any case, work efficiency could be improved if technology can be developed that allows simultaneous measurement of clear images of the dorsal and anal fins of multiple individuals in one imaging session (e.g., if each group can be imaged in one session). Other possible advancements include simultaneously taking photos and conducting pattern recognition and computation, possibly using artificial intelligence to aid the process. Furthermore, if we could video-record all of the zebrafish’s behavior during a study and reveal the physiological state of each individual automatically, it would not only be possible to reduce the workload and improve work efficiency, but also to determine treatment effects with a high level of precision.
