# Correction: Stripe pattern differences can be used to distinguish individual adult zebrafish

**DOI:** 10.1371/journal.pone.0327684

**Published:** 2025-07-02

**Authors:** Shinichi Meguro, Takahiro Hasumura

The first author, Shinichi Meguro, is incorrectly noted as the corresponding author. The correct corresponding author is Takahiro Hasumura. Takahiro Hasumura’s email address is: hasumura.takahiro@kao.com.
